# Comparison of Postoperative Complications After Gastrectomy for Gastric Cancer with Antecolic Versus Retrocolic Reconstruction: A Population-Based Study

**DOI:** 10.1245/s10434-024-15415-5

**Published:** 2024-05-15

**Authors:** Anna Junttila, Olli Helminen, Mika Helmiö, Heikki Huhta, Aapo Jalkanen, Raija Kallio, Vesa Koivukangas, Arto Kokkola, Simo Laine, Elina Lietzen, Johanna Louhimo, Sanna Meriläinen, Vesa-Matti Pohjanen, Tuomo Rantanen, Ari Ristimäki, Jari V. Räsänen, Juha Saarnio, Eero Sihvo, Vesa Toikkanen, Tuula Tyrväinen, Antti Valtola, Joonas H. Kauppila, on behalf of the FINEGO Group

**Affiliations:** 1 Division of Digestive Surgery and Urology, Turku University Hospital, Turku, Finland; 2 Surgery Research Unit, Medical Research Center Oulu, Oulu University Hospital and University of Oulu, Oulu, Finland; 3 Department of Surgery, University of Helsinki and Helsinki University Hospital, Helsinki, Finland; 4 Department of Oncology and Haematology, Oulu University Hospital, Oulu, Finland; 5 Cancer and Translational Medicine Research Unit, Medical Research Center Oulu, University of Oulu and Oulu University Hospital, Oulu, Finland; 6 Department of Surgery, University of Eastern Finland and Kuopio University Hospital, Kuopio, Finland; 7 Department of Pathology, HUSLAB, HUS Diagnostic Center, Helsinki University Hospital and University of Helsinki, Helsinki, Finland; 8 Applied Tumor Genomics Research Program, Research Programs Unit, Faculty of Medicine, University of Helsinki, Helsinki, Finland; 9 Department of General Thoracic and Oesophageal Surgery, Heart and Lung Centre, University of Helsinki and Helsinki University Hospital, Helsinki, Finland; 10 Department of Surgery, Central Finland Central Hospital, Jyväskylä, Finland; 11 Department of Cardiothoracic Surgery, Heart Center, Tampere University Hospital and University of Tampere, Tampere, Finland; 12 Department of Gastroenterology and Alimentary Tract Surgery, Tampere University Hospital, Tampere, Finland; 13 Upper Gastrointestinal Surgery, Department of Molecular Medicine and Surgery, Karolinska Institutet and Karolinska University Hospital, Stockholm, Sweden

**Keywords:** Antecolic, Retrocolic, Gastric cancer, Gastrectomy

## Abstract

**Background:**

The purpose of this study was to examine the rates of 90-day anastomotic complications and other postoperative complications after total or partial gastrectomy with antecolic versus retrocolic reconstruction in a population-based setting.

**Methods:**

This population-based nationwide retrospective cohort study included all patients undergoing total or partial gastrectomy for gastric adenocarcinoma in Finland in 2005–2016, with follow-up until 31 December 2019. Logistic regression provided odds ratios (ORs) with 95% confidence intervals (CIs) of 90-day mortality. Results were adjusted for age, sex, year of the surgery, comorbidities, tumor locations, pathological stage, and neoadjuvant therapy.

**Results:**

A total of 2063 patients having gastrectomy with antecolic (*n* = 814) or retrocolic (*n* = 1249) reconstruction were identified from the registries. The anastomotic complication rate was 3.8% with antecolic reconstruction and 5.0% with retrocolic reconstruction. Antecolic reconstruction was not associated with a higher risk of anastomotic complications compared with retrocolic reconstruction in the adjusted analysis (OR 0.69, 95% CI 0.44–1.09) of the whole cohort or in the predefined subgroups. The reoperation rate was 8.2% with antecolic reconstruction and 7.7% with retrocolic reconstruction, without statistical significance. In subgroup analysis of total gastrectomy patients, the risk of major complications was lower with antecolic reconstruction compared with retrocolic reconstruction (OR 0.62, 95% CI 0.45–0.86).

**Conclusions:**

The rate of anastomotic complications did not differ after antecolic versus retrocolic reconstruction after total or partial gastrectomy. In total gastrectomies, the risk of major complications was lower after antecolic compared with retrocolic reconstruction.

Among all cancers worldwide, gastric cancer is ranked fifth in incidence (5.6%) and fourth (7.7%) in cancer-related mortality.^1^ The surgical treatment of gastric cancer has progressed remarkably in the past decades. The proportion of endoscopic resections has increased in early-stage tumors, and multimodal therapy is the standard treatment for locally advanced tumors.^2,3^ Furthermore, gastric cancer surgery is nowadays increasingly performed using a minimally invasive technique, which has been shown to be equivalent in overall short-term morbidity and mortality compared with the open approach for locally advanced gastric cancer.^2,4^

Roux-en-Y reconstruction is a simple and robust form of reconstruction performed after a total or partial gastrectomy.^3,5^ These days, it is widely and most commonly used and has increasingly replaced the Billroth II reconstruction technique that has been used after partial gastrectomy.^3^ A recent meta-analysis of 1369 patients (Roux-en-Y, *n* = 732; and Billroth II, *n* = 637) showed that Roux-en-Y reconstruction does not increase postoperative complications but can improve postoperative quality of life owing to less remnant gastritis, dumping symptoms, reflux symptoms and esophagitis compared with Billroth II reconstruction.^6^ However, reconstruction after open total or partial gastrectomy has often been performed via the retrocolic route since it results in less tension due to the shorter route when compared with antecolic reconstruction. After implementation of laparoscopic surgery, the antecolic route has gained popularity due to its technical simplicity and is currently also more commonly used in the open approach.^7^

Thus far, the studies comparing antecolic and retrocolic reconstruction have mainly focused on patients undergoing bariatric surgery or pancreaticoduodenectomy. A recent review of 818 patients comparing antecolic and retrocolic reconstruction after partial pancreaticoduodenectomy did not reveal any relevant differences between these techniques in delayed gastric emptying or other morbidities (e.g. hemorrhage, intra-abdominal fluid collection, abscess, or reoperation rate) or mortality.^8^ A study of 138 distal gastrectomy patients showed less delayed gastric emptying with antecolic reconstruction compared with retrocolic reconstruction;^9^ however, there is a lack of large, population-based studies comparing postoperative complications after total or partial gastrectomy with antecolic versus retrocolic reconstruction for gastric cancer.

The aim of this study was to examine 90-day anastomotic complications and other postoperative complications after total or partial gastrectomy with antecolic versus retrocolic reconstruction in a population-based setting.

## Methods

### Study Design

This was a population-based, nationwide, retrospective cohort study from Finland including total or partial gastrectomy for gastric adenocarcinoma. Patients with other histological types of gastric malignancies were excluded because they were not comparable in terms of treatment and prognosis. The study period was from 1 January 2005 to 31 December 2016, with follow-up until 31 December 2019.^10^ The patients undergoing total or partial gastrectomy were compared according to 90-day postoperative complications and reoperations. The study was approved by the Regional Ethical Review Board in Oulu, Finland, the Finnish national health officials and hospital districts.^11^

## Data Collection

Retrospective comparison of different surgical operations is prone to bias in single-center studies. The Finnish National Esophago-Gastric Cancer Cohort (FINEGO) includes all patients with esophageal and gastric cancer diagnosed in Finland between 1987 and 2016.^11^ The FINEGO database contains information from the Finnish Cancer Registry, Finnish National Institute for Health and Welfare Registries, Care Register for Healthcare, and Hospital Discharge Registry. The Finnish Cancer Registry and Hospital Discharge Registry were 87% and 92.7% complete for gastric cancer, respectively.^12^ Surgically treated patients were identified using Nordic Medico-Statistical Committee (NOMESCO) surgical codes. The identification using both registries by searching for cancer diagnoses and operation codes allows almost 100% completeness on eligible patient identification. After identification of cases, available information including age, sex, comorbidity,^13^ surgery, and other variables were collected from the Finnish Cancer Registry, Finnish National Institute for Health and Welfare Registries, and Care Register for Healthcare and Hospital Discharge Registry.^11^ Medical reports were obtained from the respective healthcare units and reviewed by specialized surgeons, providing accurate information on type of resection, tumor location, histology, stage, neoadjuvant treatment and postoperative complications. All-cause mortality data were obtained from the 100% complete death registry, held by Statistics Finland until 31 December 2019.^14^

## Exposures

The study exposure group included patients undergoing total or partial gastrectomy with antecolic reconstruction, while those undergoing total or partial gastrectomy with retrocolic reconstruction were included in the control group.

## Outcomes

The primary outcome of the study was to evaluate 90-day anastomotic complication rates after total or partial gastrectomy with antecolic versus retrocolic reconstruction, while the secondary outcome included other 90-day postoperative complications (bleeding, small bowel obstruction, ileus, delayed conduit emptying, pancreatic fistula, intra-abdominal abscess, major complication, and reoperation) after total or partial gastrectomy with antecolic versus retrocolic reconstruction, according to the definitions from the Esophagectomy Complications Consensus Group (ECCG).^15^ Major complications were defined as at least Clavien–Dindo stage IIIa complications (i.e. any surgical, endoscopic, or radiological intervention, life-threatening complication requiring intensive care management, or death).^16^

## Statistical Analysis

The analyses followed a detailed a priori study protocol. For all analyses, IBM SPSS v26.0 statistical software (IBM Corporation, Armonk, NY, USA) was used. Follow-up times were calculated from the date of surgery until the time of death or the end of follow-up, whichever occurred first. Survival was calculated using the life-table method, visualized with Kaplan–Meier curves. Logistic regression provided odds ratios (ORs) with 95% confidence intervals (CIs). To avoid confounding, adjustments for seven known prognostic factors were made: age (continuous), sex (male/female), year of the surgery (continuous), comorbidity (Charlson Comorbidity Index^13^ 0, 1 or ≥2 [excluding the gastric cancer under treatment]), tumor location (proximal, middle, distal), pathological stage (stage 0–I, II, III, IV, according to 8th edition of the American Joint Committee on Cancer/Union for International Cancer Control [AJCC/UICC] staging of gastric cancer^17^) and neoadjuvant therapy (yes/no). Analyses for three subgroups were performed: (1) R0 resections; (2) total gastrectomies; and (3) distal gastrectomies. The adjustments for the subgroups were also performed as described above.

## Results

### Patients

A total of 2196 patients who underwent gastrectomy for gastric adenocarcinoma during 2005–2016 were identified from the registries. Patients operated with proximal gastrectomy (*n* = 24) or wedge resections (*n* = 6) were excluded. Antecolic or retrocolic reconstruction was performed on 2063 patients. Of the patients, 94 (4.6%) had anastomotic complications and 163 (7.9%) needed reoperation. The majority of the study patients were male and had pathological stage III disease. R0 resection was achieved in 72.4% of patients. Patient characteristics are presented in Table 1.

**Table 1:** Patient characteristics in 2063 gastrectomy patients operated with antecolic or retrocolic reconstruction for gastric adenocarcinoma in Finland 2005–2016.

	Whole cohort [*n* = 2063]	Antecolic reconstruction [*n* = 814]	Retrocolic reconstruction [*n* = 1249]
*Year of the operation*			
2005–2008	802 (38.9)	328 (40.3)	474 (38.0)
2009–2012	680 (33.0)	253 (31.1)	427 (34.2)
2013–2016	581 (28.2)	233 (28.6)	348 (27.9)
Age, years [median (IQR)]	69 (62–78)	70 (63–79)	69 (62–78)
*Sex*			
Male	1154 (55.9)	455 (55.9)	699 (56.0)
Female	909 (44.1)	359 (44.1)	550 (44.0)
*Charlson comorbidity index*			
0	1042 (50.5)	407 (50.0)	635 (50.8)
1	623 (30.2)	248 (30.5)	375 (30.0)
≥2	398 (19.3)	159 (19.5)	239 (19.2)
*Tumor location*			
Proximal, including cardia	237 (11.5)	87 (10.7)	150 (12.0)
Middle/body	866 (42.0)	331 (40.7)	535 (42.8)
Distal	959 (46.5)	396 (48.6)	563 (45.1)
*Neoadjuvant treatment*			
Yes	288 (14.0)	72 (8.9)	216 (17.3)
No	1768 (85.7)	740 (90.9)	1028 (82.3)
Missing	7 (0.3)	2 (0.2)	5 (0.4)
*Pathological stage*			
0–I	502 (24.3)	184 (22.6)	318 (25.5)
II	590 (28.6)	244 (30.0)	346 (27.7)
III	720 (34.9)	271 (33.3)	449 (35.9)
IV	214 (10.4)	94 (11.5)	120 (9.6)
Missing	37 (1.8)	21 (2.6)	16 (1.3)
*Resection type*			
Total gastrectomy	1265 (61.3)	474 (58.2)	791 (63.3)
Distal gastrectomy	798 (38.7)	340 (41.8)	458 (36.7)
*Operative approach*			
Open	1965 (95.2)	735 (90.3)	1230 (98.5)
Laparoscopic	98 (4.8)	79 (9.7)	19 (1.5)
*Anastomosis*			
Handsewn	446 (21.6)	124 (15.3)	322 (25.8)
Stapled	1615 (78.3)	688 (84.7)	927 (74.2)
*Lymphadenectomy*			
D0	287 (13.9)	151 (18.6)	136 (10.9)
D1	964 (46.7)	447 (54.9)	517 (41.4)
D2	751 (36.4)	201 (24.7)	550 (44.0)
Missing	61 (3.0)	15 (1.8)	46 (3.7)
*Radicality*			
R0	1493 (72.4)	568 (69.7)	925 (74.1)
R1	171 (8.3)	65 (8.0)	106 (8.5)
R2	156 (7.6)	81 (10.0)	75 (6.0)
Palliative intent	160 (7.8)	81 (10.0)	79 (6.3)
Missing	83 (4.0)	19 (2.3)	64 (5.1)
9*0-day complications*			
Anastomotic complications	94 (4.6)	31 (3.8)	63 (5.0)
Bleeding	76 (3.7)	36 (4.4)	40 (3.2)
Small bowel obstruction	20 (1.0)	6 (0.7)	14 (1.1)
Ileus	100 (4.8)	43 (5.3)	57 (4.6)
Delayed conduit emptying	35 (1.7)	12 (1.5)	23 (1.8)
Pancreatic fistula	15 (0.7)	2 (0.2)	13 (1.0)
Intra-abdominal abscess	157 (7.6)	50 (6.1)	107 (8.6)
Major complication	345 (16.7)	125 (15.4)	220 (17.6)
Reoperation	163 (7.9)	67 (8.2)	96 (7.7)

Data are expressed as *n* (%) unless otherwise specified

*IQR* interquartile range

## Primary Outcomes

The anastomotic complication rate was 3.8% with antecolic reconstruction and 5.0% with retrocolic reconstruction. Antecolic reconstruction was not associated with a higher risk of anastomotic complications compared with retrocolic reconstruction in the crude (OR 0.75, 95% CI 0.48–1.16) or adjusted analyses (OR 0.69, 95% CI 0.44–1.09) of the whole cohort (Table 2) or in the predefined subgroups (Tables 3, 4 and 5).

**Table 2:** Ninety-day complications after total or partial gastrectomy operated with antecolic or retrocolic reconstruction for gastric adenocarcinoma, expressed as odds ratios with 95% confidence intervals

	No. of patients [*n *= 2063]	Antecolic reconstruction [*n* = 814]	Retrocolic reconstruction [*n* = 1249]
*Anastomotic complication*			
All patients (crude)	2063	0.75 (0.48–1.16)	1.00 (Reference)
All patients (adjusted)^a^	2063	0.69 (0.44–1.09)	1.00 (Reference)
*Bleeding*			
All patients (crude)	2063	1.40 (0.88–2.21)	1.00 (Reference)
All patients (adjusted)^a^	2063	1.45 (0.90–2.31)	1.00 (Reference)
*Small bowel obstruction*			
All patients (crude)	2063	0.66 (0.25–1.71)	1.00 (Reference)
All patients (adjusted)^a^	2063	0.63 (0.24–1.65)	1.00 (Reference)
*Ileus*			
All patients (crude)	2063	1.17 (0.78–1.75)	1.00 (Reference)
All patients (adjusted)^a^	2063	1.14 (0.75–1.73)	1.00 (Reference)
*Delayed conduit emptying*			
All patients (crude)	2063	0.80 (0.40–1.61)	1.00 (Reference)
All patients (adjusted)^a^	2063	0.67 (0.32–1.38)	1.00 (Reference)
*Pancreatic fistula*			
All patients (crude)	2063	0.23 (0.05–1.04)	1.00 (Reference)
All patients (adjusted)^a^	2063	0.25 (0.06–1.14)	1.00 (Reference)
*Intra-abdominal abscess*			
All patients (crude)	2063	0.70 (0.49–1.00)	1.00 (Reference)
All patients (adjusted)^a^	2063	0.72 (0.50–1.02)	1.00 (Reference)
*Major complication*			
All patients (crude)	2063	0.85 (0.67–1.08)	1.00 (Reference)
All patients (adjusted)^a^	2063	0.84 (0.66–1.08)	1.00 (Reference)
*Reoperation*			
All patients (crude)	2063	1.08 (0.78–1.49)	1.00 (Reference)
All patients (adjusted)^a^	2063	1.02 (0.73–1.43)	1.00 (Reference)

^a^Adjustment for age (continuous), sex (male/female), year of the surgery (continuous), Charlson Comorbidity Index (0, 1 or ≥2 [excluding the gastric cancer under treatment]), tumor location (proximal, middle, distal), pathological stage (0–I, II, III, IV), and neoadjuvant therapy (yes/no)

**Table 3:** Ninety-day complications after R0 resection gastrectomy operated with antecolic or retrocolic reconstruction for gastric adenocarcinoma, expressed as odds ratios with 95% confidence intervals

	No. of patients [*n* = 1493]	Antecolic reconstruction [*n* = 568]	Retrocolic reconstruction [*n* = 925]
*Anastomotic complication*			
All patients (crude)	1493	0.81 (0.48–1.36)	1.00 (Reference)
All patients (adjusted)^a^	1493	0.80 (0.47–1.36)	1.00 (Reference)
*Bleeding*			
All patients (crude)	1493	1.26 (0.72–2.19)	1.00 (Reference)
All patients (adjusted)^a^	1493	1.28 (0.73–2.26)	1.00 (Reference)
*Small bowel obstruction*			
All patients (crude)	1493	0.41 (0.86–1.91)	1.00 (Reference)
All patients (adjusted)^a^	1493	0.38 (0.08–1.82)	1.00 (Reference)
*Ileus*			
All patients (crude)	1493	0.91 (0.54–1.53)	1.00 (Reference)
All patients (adjusted)^a^	1493	0.92 (0.53–1.57)	1.00 (Reference)
*Delayed conduit emptying*			
All patients (crude)	1493	0.77 (0.35–1.71)	1.00 (Reference)
All patients (adjusted)^a^	1493	0.69 (0.31–1.55)	1.00 (Reference)
*Pancreatic fistula*			
All patients (crude)	1493	0.27 (0.03–2.25)	1.00 (Reference)
All patients (adjusted)^a^	1493	0.26 (0.03–2.28)	1.00 (Reference)
*Intra-abdominal abscess*			
All patients (crude)	1493	0.58 (0.38–0.89)	1.00 (Reference)
All patients (adjusted)^a^	1493	0.61 (0.40–0.93)	1.00 (Reference)
*Major complication*			
All patients (crude)	1493	0.73 (0.55–0.98)	1.00 (Reference)
All patients (adjusted)^a^	1493	0.74 (0.55–1.00)	1.00 (Reference)
*Reoperation*			
All patients (crude)	1493	1.02 (0.69–1.53)	1.00 (Reference)
All patients (adjusted)^a^	1493	1.02 (0.68–1.54)	1.00 (Reference)

^a^Adjustment for age (continuous), sex (male/female), year of the surgery (continuous), Charlson Comorbidity Index (0, 1 or ≥2 [excluding the gastric cancer under treatment]), tumor location (proximal, middle, distal), pathological stage (0–I, II, III, IV), and neoadjuvant therapy (yes/no)

**Table 4:** Ninety-day complications in patients undergoing total gastrectomy with antecolic or retrocolic reconstruction for gastric adenocarcinoma, expressed as odds ratios with 95% confidence intervals

	No. of patients [*n* = 1265]	Antecolic reconstruction [*n* = 474]	Retrocolic reconstruction [*n* = 791]
*Anastomotic complication*			
All patients (crude)	1265	0.84 (0.51–1.40)	1.00 (Reference)
All patients (adjusted)^a^	1265	0.74 (0.44–1.25)	1.00 (Reference)
*Bleeding*			
All patients (crude)	1265	1.31 (0.73–2.35)	1.00 (Reference)
All patients (adjusted)^a^	1265	1.29 (0.71–2.34)	1.00 (Reference)
*Small bowel obstruction*			
All patients (crude)	1265	0.18 (0.02–1.45)	1.00 (Reference)
All patients (adjusted)^a^	1265	0.21 (0.03–1.64)	1.00 (Reference)
*Ileus*			
All patients (crude)	1265	0.65 (0.35–1.19)	1.00 (Reference)
All patients (adjusted)^a^	1265	0.64 (0.34–1.19)	1.00 (Reference)
*Delayed conduit emptying*			
All patients (crude)	1265	0.52 (0.19–1.42)	1.00 (Reference)
All patients (adjusted)^a^	1265	0.38 (0.13–1.17)	1.00 (Reference)
*Pancreatic fistula*			
All patients (crude)	1265	0.14 (0.02–1.06)	1.00 (Reference)
All patients (adjusted)^a^	1265	0.12 (0.02–0.92)	1.00 (Reference)
*Intra-abdominal abscess*			
All patients (crude)	1265	0.66 (0.42–1.02)	1.00 (Reference)
All patients (adjusted)^a^	1265	0.65 (0.41–1.02)	1.00 (Reference)
*Major complication*			
All patients (crude)	1265	0.64 (0.47–0.87)	1.00 (Reference)
All patients (adjusted)^a^	1265	0.62 (0.45–0.86)	1.00 (Reference)
*Reoperation*			
All patients (crude)	1265	0.84 (0.56–1.27)	1.00 (Reference)
All patients (adjusted)^a^	1265	0.77 (0.50–1.18)	1.00 (Reference)

^a^Adjustment for age (continuous), sex (male/female), year of the surgery (continuous), Charlson Comorbidity Index (0, 1 or ≥2 [excluding the gastric cancer under treatment]), tumor location (proximal, middle, distal), pathological stage (0–I, II, III, IV), and neoadjuvant therapy (yes/no)

**Table 5:** Ninety-day complications in patients undergoing distal gastrectomy with antecolic or retrocolic reconstruction for gastric adenocarcinoma, expressed as odds ratios with 95% confidence intervals

	No. of patients [*n* = 798]	Antecolic reconstruction [*n* = 340]	Retrocolic reconstruction [*n* = 458]
*Anastomotic complication*			
All patients (crude)	1265	0.58 (0.24–1.43)	1.00 (Reference)
All patients (adjusted)^a^	1265	0.51 (0.20–1.29)	1.00 (Reference)
*Bleeding*			
All patients (crude)	1265	1.58 (0.74–3.37)	1.00 (Reference)
All patients (adjusted)^a^	1265	1.71 (0.78–3.78)	1.00 (Reference)
*Small bowel obstruction*			
All patients (crude)	1265	1.35 (0.39–4.71)	1.00 (Reference)
All patients (adjusted)^a^	1265	1.26 (0.35–4.51)	1.00 (Reference)
*Ileus*			
All patients (crude)	1265	2.07 (1.14–3.78)	1.00 (Reference)
All patients (adjusted)^a^	1265	2.21 (1.16–4.18)	1.00 (Reference)
*Delayed conduit emptying*			
All patients (crude)	1265	1.35 (0.47–4.00)	1.00 (Reference)
All patients (adjusted)^a^	1265	1.08 (0.35–3.36)	1.00 (Reference)
*Pancreatic fistula*			
All patients (crude)	1265	1.35 (0.08–21.63)	1.00 (Reference)
All patients (adjusted)^a^	1265	1.00 (0.04–23.84)	1.00 (Reference)
*Intra-abdominal abscess*			
All patients (crude)	1265	0.81 (0.45–1.43)	1.00 (Reference)
All patients (adjusted)^a^	1265	0.82 (0.45–1.48)	1.00 (Reference)
*Major complication*			
All patients (crude)	1265	1.37 (0.93–2.01)	1.00 (Reference)
All patients (adjusted)^a^	1265	1.35 (0.90–2.02)	1.00 (Reference)
*Reoperation*			
All patients (crude)	1265	1.85 (1.04–3.28)	1.00 (Reference)
All patients (adjusted)^a^	1265	1.79 (0.98–3.28)	1.00 (Reference)

^a^Adjustment for sex, Charlson Comorbidity Index (0, 1 or ≥2 [excluding the gastric cancer under treatment]), tumor location (proximal, middle, distal), pathological stage (0–I, II, III, IV) and neoadjuvant therapy (yes/no)

## Secondary Outcomes

The rates of all 90-day complications are presented in Table 1. The reoperation rate was 7.9% in the whole cohort, 8.2% with antecolic reconstruction, and 7.7% with retrocolic reconstruction. No statistically significant differences between antecolic versus retrocolic reconstruction according to reoperations were found in the crude or adjusted model of the whole cohort or the subgroups.

In the whole cohort, antecolic reconstruction was associated with a lower risk of intra-abdominal abscess in the crude analysis (OR 0.70, 95% CI 0.49–1.00) compared with retrocolic reconstruction, while no statistical significance was found in the adjusted model (OR 0.72, 95% CI 0.72–1.02) [Table 2]. No association was found between reconstruction type and bleeding, small bowel obstruction, ileus, delayed conduit emptying, pancreatic fistula, or major complications (Table 2).

In the subgroup analysis of R0 resected patients, antecolic reconstruction was associated with a lower risk of intra-abdominal abscess in the crude (OR 0.58, 95% CI 0.38–0.89) and adjusted analysis (OR 0.61, 95% CI 0.40–0.93) [Table 3] compared with retrocolic reconstruction. The risk of major complications was lower in the crude analysis (OR 0.73, 95% CI 0.55–0.98), while no statistical significance was found in the adjusted analysis (OR 0.74, 95% CI 0.74–1.00) [Table 3]. Furthermore, no association was found between reconstruction type and bleeding, small bowel obstruction, ileus, delayed conduit emptying, or pancreatic fistula.

With patients undergoing total gastrectomy, the rate of major complications was lower with antecolic reconstruction in the crude (OR 0.64, 95% CI 0.47–0.87) and adjusted analyses (OR 0.62, 95% CI 0.45–0.86) [Table 4] compared with retrocolic reconstruction. Moreover, the risk of pancreatic fistula was lower with antecolic reconstruction in the adjusted analysis (OR 0.12, 95% CI 0.02–0.92) [Table 4] compared with retrocolic reconstruction. However, no association was found between reconstruction type and bleeding, small bowel obstruction, ileus, delayed conduit emptying, or intra-abdominal abscesses.

No statistically significant differences between reconstruction type and any 90-day postoperative complications were found in the crude or adjusted analysis in the subgroup of patients undergoing distal gastrectomy (Table 5). The observed 90-day survival was 92.5% with antecolic reconstruction and 93.3% with retrocolic reconstruction (*p* = 0.521).

## Discussion

This population-based nationwide cohort study suggests no difference in anastomotic complications or reoperations comparing antecolic reconstruction with retrocolic reconstruction after total or partial gastrectomy for gastric cancer. No statistically significant differences were found in the adjusted model or in subgroup analysis between the two reconstruction techniques. In the subgroup analysis of patients undergoing total gastrectomy, the risk of major complications was lower with antecolic reconstruction compared with retrocolic reconstruction.

The main strength of this study was its population-based setting to avoid selection bias. The study analysis was performed according to an *a priori* study protocol to minimize the risk of chance findings. Furthermore, a major strength is the complete identification and 100% complete follow-up of all study patients diagnosed with gastric adenocarcinoma in Finland. The Finnish national registries are based on independent and automatic reporting of diagnosis and procedure codes from the hospitals, to hospital discharge registry and also clinicians reporting new cancer cases, enabling dependable patient identification with high coverage.^12^ The complication data were collected and categorized comprehensively by specialized surgeons, which increases the quality of the present study. On the other hand, due to the retrospective nature of this study, there is a possibility that some complications may have been missed during the review of patient records. The results were adjusted for known potential confounders, while some unknown bias or confounding may have occurred due to the observational setting of the study. The sample size of the FINEGO database was considered sufficient to also enable analyses in smaller subgroups.

To our knowledge, this is the first study comparing 90-day complication rates after total or partial gastrectomy with antecolic versus retrocolic reconstruction for gastric cancer in a population-based nationwide setting. In this study cohort, the anastomotic complication rate was 3.8% with antecolic reconstruction and 5.0% with retrocolic reconstruction. The reconstruction type was not associated with a risk of anastomotic complications in the main or subgroup analyses. A systematic review and meta-analysis of 1161 patients undergoing distal gastrectomy for gastric cancer with Billroth I, Billroth II, or Roux-en-Y reconstruction did not find any association between reconstruction technique and anastomotic leakage;^18^ however, the study did not report the reconstruction route used. It has been speculated that more tension in the anastomotic area with antecolic reconstruction increases the risk of anastomotic complications.^5,7^ After implementation of a laparoscopic approach in gastric cancer surgery, the popularity of antecolic reconstruction has increased due to its technical simplicity and quickness compared with retrocolic reconstruction.^7^ A systematic review and meta-analysis of randomized controlled trials (a total of 26 RCTs with 8301 patients) did not find significant differences in anastomotic leakages between the laparoscopic and open approaches.^19^ The rate of anastomotic leakage was 1.7% in patients undergoing laparoscopic total gastrectomy with antecolic Roux-en-Y reconstruction compared with a rate of 4.2% after open total gastrectomy in the series of 329 gastric cancer patients in a single-center study.^20^ Furthermore, antecolic reconstruction was not associated with anastomotic leakages in the meta-analysis of 13,660 patients undergoing laparoscopic Roux-en-Y gastric bypass.^21^ Taken together, our findings are in line with earlier published studies showing no association between anastomotic complications and reconstruction technique.

Gastric cancer surgery is conventionally connected to high rates of postoperative complications ranging from 9 to 46%;^22–24^ however, the reconstruction route is very rarely reported in gastric cancer studies. In the meta-analysis of patients undergoing laparoscopic Roux-en-Y gastric bypass, the incidence of total complications was 10.8% with antecolic reconstruction and 11.2% with retrocolic reconstruction.^21^ A study of 152 patients undergoing curative intent total or distal gastrectomy for gastric cancer found extended lymphadenectomy as a risk factor for postoperative intra-abdominal infectious complications in univariate analyses but not in multivariate analyses.^25^ Furthermore, high incidence rates of postoperative pancreatic fistula have been reported, especially after total gastrectomy with extended lymph node dissection.^26^ In our study, the rate of major complications was 15.4% after antecolic reconstruction and 17.6% after retrocolic reconstruction. A lower risk of major complications and pancreatic fistula was seen after total gastrectomy with antecolic reconstruction compared with retrocolic reconstruction, suggesting a higher risk of pancreatic injury when creating retrocolic reconstruction. Speculatively, the lack of manipulation of the colonic mesentery and the retroperitoneum in an antecolic reconstruction could lead to reduced complications; however, the study results describe an association, not causation, and prospective and larger sample size studies are required for definitive conclusions.

The 90-day mortality rates after total or partial gastrectomies have varied from 4.6% to 16.0% in previous studies.^27,28^ In our study, the 90-day mortality rate was 7.5% with antecolic reconstruction and 6.7% with retrocolic reconstruction, without statistical significance. In the present study, the follow-up for complications was limited to 90 days. It is known that, for example, internal hernias are rare in the early postoperative phase and occur mostly within 2 years of surgery.^29^ The retrocolic reconstruction route has been considered as a risk factor for internal hernias due to more possible hernia sites and also including the most common site of internal hernia through defect of the transverse mesocolon, and therefore antecolic reconstruction should be favored.^29,30^ In our study, antecolic reconstruction was not associated with a higher risk of anastomotic complications, and it suggested a decreased risk of major complications and pancreatic fistula after total gastrectomy compared with retrocolic reconstruction. Due to these findings and the technical simplicity of antecolic reconstruction, this method should be the preferred reconstruction route.

## Conclusion

In this population-based nationwide study, no difference in the rate of anastomotic complications was seen after antecolic versus retrocolic reconstruction after total or partial gastrectomy for gastric cancer.
